# Intergenerational transmission of comorbid internalizing and externalizing psychopathology at age 11: Evidence from an adoption design for general transmission of comorbidity rather than homotypic transmission

**DOI:** 10.1017/S0954579424000968

**Published:** 2024-10-04

**Authors:** Kristine Marceau, Sohee Lee, Muskan Datta, Olivia C. Robertson, Daniel S. Shaw, Misaki N. Natsuaki, Leslie D. Leve, Jody M. Ganiban, Jenae M. Neiderhiser

**Affiliations:** 1 Department of Human Development and Family Science, Purdue University, West Lafayette, IN, USA; 2 Department of Epidemiology and Biostatistics, School of Public Health, Indiana University, Bloomington, IN, USA; 3 Department of Psychology, University of Pittsburgh, Pittsburgh, PA, USA; 4 Department of Psychology, University of California, Riverside, CA, USA; 5 Prevention Science Institute, University of Oregon, Eugene, OR, USA; 6 Department of Psychology, George Washington University, WashingtonWA, DC, USA; 7 Department of Psychology, Penn State University, University Park, PA, USA

**Keywords:** adoption design, comorbidity, intergenerational transmission, internalizing and externalizing, severity and directionality

## Abstract

Psychopathology is intergenerationally transmitted through both genetic and environmental mechanisms via heterotypic (cross-domain), homotypic (domain-specific), and general (e.g., “p-factor”) pathways. The current study leveraged an adopted-at-birth design, the Early Growth and Development Study (57% male; 55.6% White, 19.3% Multiracial, 13% Black/African American, 10.9% Hispanic/Latine) to explore the relative influence of these pathways via associations between adoptive caregiver psychopathology (indexing potential environmental transmission) and birth parent psychopathology (indexing genetic transmission) with adolescent internalizing and externalizing symptoms. We included composite measures of adoptive and birth parent internalizing, externalizing, and substance use domains, and a general “p-factor.” Age 11 adolescent internalizing and externalizing symptom scores were the average of adoptive parent reports on the Child Behavior Checklist (*n* = 407). Examining domains independently without addressing comorbidity can lead to incorrect interpretations of transmission mode. Therefore, we also examined symptom severity (like the “p-factor”) and an orthogonal symptom directionality score to more cleanly disentangle transmission modes. The pattern of correlations was consistent with mostly general transmission in families with youth showing comorbid internalizing and externalizing symptoms, rather than homotypic transmission. Findings more strongly supported potential environmental or evocative mechanisms of intergenerational transmission than genetic transmission mechanisms (though see limitations). Parent-specific effects are discussed.

## Introduction

The goal of decades of research into the intergenerational transmission of internalizing and externalizing psychopathology has been to identify the mechanisms by which psychopathology runs in families in order to break the cycle for future generations. Drawing from the developmental psychopathology framework (e.g., Cicchetti, 2016), the present study is grounded by the premise that intergenerational transmission of psychopathology includes both equifinality (similar outcomes can emerge from a diverse set of risks) and multifinality (multiple outcomes can emerge from the same set of risks) and reflects interplay between genetic and environmental influences. A large literature now shows that intergenerational transmission of psychopathology includes both genetic and environmental components, occurs via direct (e.g., effects of one person’s psychopathology on the others’) and indirect (e.g., via differences in parenting) mechanisms, and runs in both directions: parent-to-child and child-to-parent (Jami et al., 2021; Marceau et al., 2022). Further, intergenerational transmission of psychopathology may occur via homotypic, heterotypic, and general transmission.

**Figure 1.**
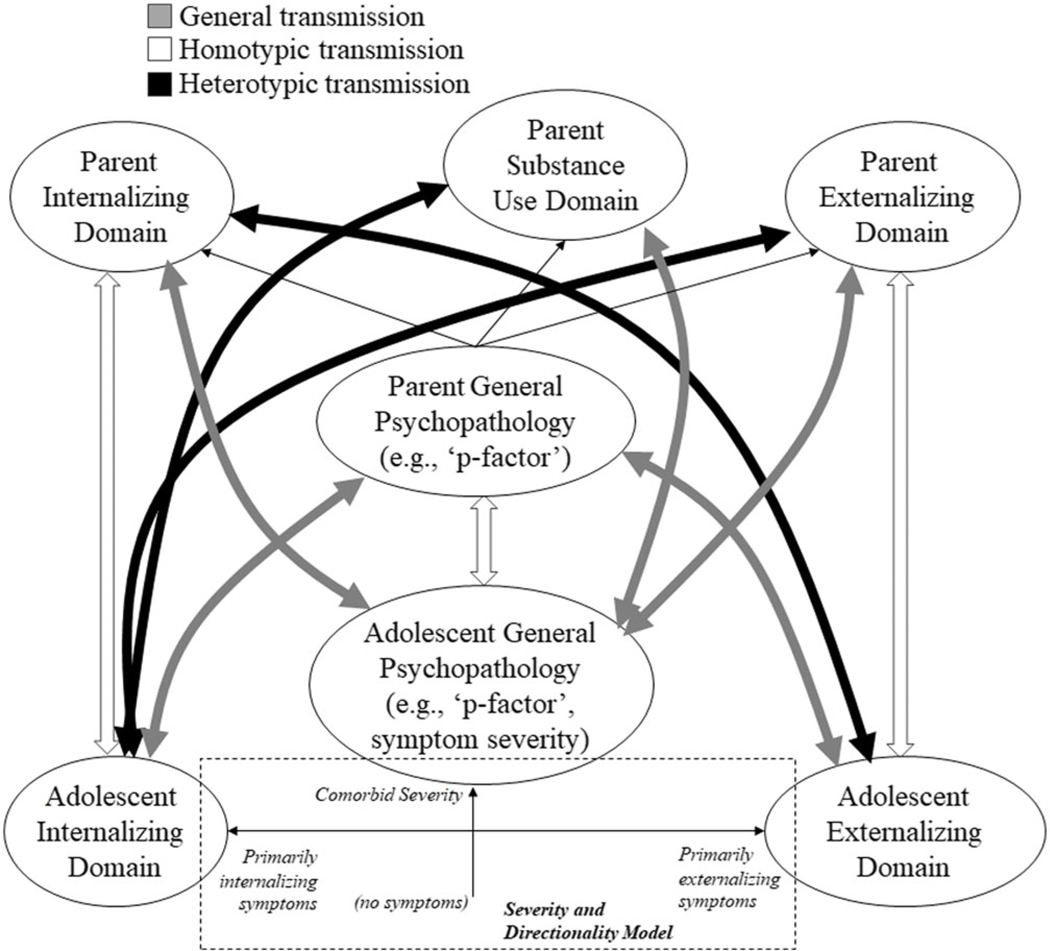
General, homotypic, and heterotypic intergenerational transmission. Parent psychopathology domains are depicted along the top and adolescent psychopathology domains across the bottom. White-filled arrows denote homotypic transmission (domain-specific), black-filled arrows depict heterotypic (cross-domain) transmission, and gray-filled arrows depict general transmission (e.g., transmission that operates through a general factor). The white-filled arrow between the “p-factor” constructs across generations reflects the potential of homotypic transmission of comorbidity. All filled arrows are depicted as bi-directional, as they may be further specified into directional arrows from parents to youth that reflect inherited genetic influences (in the present study tested via associations with birth parent psychopathology), directional arrows from parents to youth that reflect environmental influences (in the present study, supported though not directly tested by associations with adoptive parent psychopathology), or directional arrows from youth to parents that reflect evocative influences (in the present study, another explanation for associations with adoptive parent psychopathology). The hashed box depicts the severity-directionality model, wherein the *y*-axis is symptom severity, and high scores reflect the adolescent general psychopathology comorbid symptom severity, and the *x*-axis is symptom directionality, such that high directionality = more pure externalizing, low directionality = more pure internalizing, reflecting the domain scores after accounting for/independent of symptom severity. Associations with symptom severity and directionality are interpreted as homotypic, heterotypic, or general based on which parent score (e.g, which domain, or the general p-factor) is correlated with it, as described in Figures 3–6.

Homotypic transmission refers to psychopathology surfacing in the same form across generations (e.g., parental internalizing problems and child internalizing symptoms). Heterotypic transmission refers to psychopathology surfacing in different forms across generations (e.g., parental internalizing problems but child externalizing symptoms). It has been hypothesized that heterotypic transmission may in part be driven by the transmission of a general vulnerability to psychopathology that could be expressed as internalizing or externalizing symptoms for individual family members (Marceau et al., 2022). This general vulnerability is often referred to as the p-factor, (see e.g., Caspi & Moffitt, 2018), which across multiple conceptualizations theoretically encompasses a general liability that is common to multiple forms of psychopathology (see e.g., Caspi & Moffitt, 2018; Hartman, 2021; Lahey et al., 2021). The p-factor may thus explain comorbidity across internalizing and externalizing spectra, in addition to being a mechanism by which heterotypic transmission occurs. The goal of the current study was to examine genetic and environmental mechanisms of homotypic, heterotypic, and general intergenerational transmission of psychopathology from parents to their adolescent offspring (Figure 1). We do so by assessing correlations between caregiving parent and birth parent (separate individuals in an adopted-at-birth design) psychopathology with adolescent psychopathology symptoms. Although we focus on potential environmental mechanisms captured by caregiving adoptive parents here, it is important to keep in mind that associations of caregiving adoptive parent and adolescent psychopathology likely reflect both environmental parent-to-child effects and evocative effects of the child on the parent (Jami et al., 2021).

The majority of our knowledge about genetic and environmental mechanisms of intergenerational transmission of psychopathology comes from twin studies, which have shown that both parents’ and adolescents’ genetics and environments each account for part of the correlation between parent and adolescent psychopathology (Marceau & Neiderhiser, 2022; Marceau et al., 2022). Genetically informed designs including twin and adoption studies provide evidence that depression in parents, especially mothers, is more likely to operate as an environmental risk than a genetic one (Natsuaki et al., 2014). These findings are generally replicated for general internalizing symptom transmission (McAdams et al., 2015) and anxiety (Eley et al., 2015) as well (but see Hannigan et al., 2018 which found evidence of genetic transmission between prenatal depressive symptoms and child internalizing). For externalizing symptoms, on the other hand, there is evidence of both genetic and environmental homotypic transmission (D’Onofrio et al., 2007; Knopik et al., 2006, 2009; Koenig et al., 2010; Silberg et al., 2012; Waldron et al., 2012), although patterns differed somewhat by child sex and specific disorders measured (see Marceau et al., 2022 for review). Taken together, this body of work suggests that homotypic intergenerational transmission of internalizing symptoms is more likely to reflect environmental mechanisms, whereas the homotypic intergenerational transmission of externalizing symptoms may reflect both genetic and environmental mechanisms.

There are fewer studies that examine the genetic and environmental influences on heterotypic transmission. Current evidence suggests that this mode of transmission is more likely to reflect environmental mechanisms for associations between parent externalizing symptoms and child internalizing symptoms (Glowinski et al., 2004; Silberg et al., 2012). However, there was mixed evidence for associations between parent internalizing symptoms and child externalizing symptoms, with some studies showing genetic transmission (Silberg et al., 2010; Singh et al., 2011) and others environmental transmission (McAdams et al., 2015).

### Comorbidity

One feature of psychopathology especially prominent in adolescence is comorbidity (Beauchaine & McNulty, 2013; Caron & Rutter, 1991; Willner et al., 2014). It is important to note that general psychopathology factors, regardless of construction, capture comorbidity because to score high on a general factor, an individual must have symptoms across multiple domains. Here, we leverage the severity-directionality model which reorganizes two psychopathology domain scores (e.g., internalizing symptoms and externalizing symptoms) into two orthogonal scores (Essex et al., 2003; Marceau et al., 2022). The first score, symptom severity, is conceptually similar to a second-order “p-factor” in hierarchical models of psychopathology (Caspi & Moffitt, 2018); only youth with comorbid problems can achieve the highest scores for symptom severity (Marceau et al., 2022). The second score, symptom directionality, indexes differentiation of problem type on a continuum from more pure internalizing symptoms (highly negative values) to more pure externalizing symptoms (highly positive values). This measure of symptom differentiation is relatively novel in the field and is not directly captured by other models of psychopathology (Marceau & Neiderhiser, 2022; Marceau et al., 2022).

Inclusion of both symptom severity and directionality scores strengthen inferences about the mechanisms of intergenerational transmission of psychopathology. For example, evidence of an association between parent and child internalizing symptoms would indicate homotypic transmission. However, if internalizing symptoms are comorbid with externalizing symptoms within the parent or child, this association may reflect general psychopathology transmission mechanism as well, which would be hidden if externalizing symptoms were not also accounted for. In the case of comorbid internalizing and externalizing symptoms, we would expect to see parent internalizing symptoms predict child general severity of symptoms. If the parent-child internalizing symptom correlation reflected homotypic transmission specifically, however, the correlation of parent internalizing symptoms with general symptom severity would be weaker than the parent-child internalizing symptom correlation. The strongest inference about homotypic transmission, in this example, would be an association with directionality – specifically, more parent internalizing symptoms being correlated with a preponderance of internalizing symptoms, or in other words, symptom differentiation toward the internalizing domain in youth. Thus, comparing the effect sizes of associations across measures of internalizing symptoms, externalizing symptoms, symptom severity, and symptom directionality measures is expected to yield more comprehensive and stronger inferences about the intergenerational transmission of psychopathology by explicitly considering comorbidity.

### Present study

The Early Growth and Development Study (EGDS) uses an adopted-at-birth design wherein children were placed for adoption at birth into a family that is not genetically related to the child. This design affords a unique opportunity to examine the relative influences of heritable psychopathology risk and caregiving parent psychopathology risk for adolescent internalizing and externalizing symptoms because associations of birth parents and adopted children (who share genetic material but not the postnatal environment) reflect genetic influences, whereas associations of adoptive parents and adopted children (who share the postnatal environment but no genetics) reflect either environmental or child evocative influences. Because our measures for adoptive parent psychopathology are generally lifetime measures and occur years before the 11-year-old assessment of the adolescent, we conceptualize and interpret these findings as “potential environmental” effects of living in a home with caregiver(s) with psychopathology, which may be associated with adolescent psychopathology directly (assessed here) and/or via indirect mechanisms such as parenting (out of the scope of this study). However, evocative gene-environment correlation may also operate in adoptive families, wherein adoptive parent psychopathology could be a response to adolescents’ genetically influenced psychopathology symptoms. As a short-hand, we will refer to associations between adoptive parent and adolescent psychopathology as “environmental” because both direct environment and evocative gene-environment correlation contain an aspect of environmental influence.

In sum, we will test the role of direct genetic (birth parent psychopathology) and environmental (adoptive parent psychopathology) transmission between parents and 11-year-old youth. A major contribution of this study is that we will specifically examine general, homotypic, and heterotypic mechanisms. Based on the findings reviewed above, we also expected to see some general (e.g., Marceau et al., 2022) and some homotypic (e.g., McAdams et al., 2015; Silberg et al., 2010, 2012) transmission. We expected less evidence of heterotypic transmission primarily because cross-domain parent-child correlations are typically smaller than within-domain parent-child correlations (e.g., Silberg et al., 2010, 2012). We believe prior findings of heterotypic transmission will be better explained by general transmission when general transmission is modeled explicitly, because most studies that have examined heterotypic transmission did not account for comorbidity (see Marceau et al., 2022). Finally, based on previous findings examining symptom severity (Marceau & Neiderhiser, 2022; Marceau et al., 2022) we expected homotypic transmission of comorbidity, assessed via associations of general psychopathology scores across generations.

## Methods

### Participants and procedures

Participants were drawn from the Early Growth and Development Study (Leve et al., 2019), a prospective longitudinal study of children adopted at birth (mean age = 6 days, SD = 12.45, maximum = 91 days). In brief, families were recruited with the help of 45 adoption agencies in 15 states; inclusion criteria comprised: (1) domestic adoptions in the United States, (2) to non-relative families, (3) with placement prior to 3 months of age, (4) the child had no major medical issues, and (5) birth and adoptive parents could understand 8^th^ grade-level English. We leveraged data from 561 adopted children (57% male, 55.6% White non-Hispanic, 19.3% multiracial, 13% Black, 10.9%, Hispanic/Latine, 1.2% other) and their birth and adoptive parents, collected in two cohorts recruited in 2003–2006 (cohort 1) and 2008–2010 (cohort 2).

Adoptive family composition included mostly adoptive mother and father couples (90.8% at the start of the study, 80% at the age 11 assessment), with some same-sex couples (study start: 7.0%), and single parents (study start: 1.8 %). Some couples separated or divorced over the course of the present study (11.7% at the age 11 assessment). Adoptive parents are labeled adoptive parent 1 (AP1; 96.4% mothers) and adoptive parent 2 (AP2; 94% fathers) as self-identified at recruitment. We chose to examine AP1 and AP2 (as opposed to mothers and fathers) in order to be inclusive of families with same-sex parents while avoiding statistical problems (non-independence) that would arise by including two mothers/fathers in a single family in associations of mothers/fathers and youth. Compared to Adoptive Parents (APs), Birth Parents (BPs) were more racially and ethnically diverse (BP: ∼70% White vs. AP: ∼90% White), younger at the time of placement (mid-20s vs. the mid-late 30s), had a lower median education at the last assessment (BP: high school vs. AP: 4-year college), and had a lower median income at the last assessment (BP: below $40,000 vs. AP: between $100,000 and $150,000). Please see Leve et al. (2019) and Reiss et al. (2023) for more thorough sample descriptions and Tables. The data collection used in the current study was approved by the Institutional Review Boards at the University of Oregon and Penn State University, and use of these data for the current study was approved by Purdue University.

#### Measures

Code and results for all data preparation are available on https://osf.io/zx47e/?view_only=7729aef0ffd24322bc7132621d020a89.

**Adolescent Psychopathology** was measured using the average of AP1 and AP2 reports on the internalizing and externalizing broadband scales of the Child Behavior Checklist (Achenbach & Rescorla, 2001; Achenbach, 2011) at the age 11 assessment (*M* = 11.38, *SD* = .54, Range = 10.48–12.88) years old. Cronbach’s alphas suggested good internal consistency for each rater, *α* > .90, and AP1 and AP2 scores were correlated for internalizing symptoms, *r* = .47, *p* < .001, and externalizing symptoms, *r* = .69, *p* = <.001. We averaged AP1 and AP2 reports to obtain multi-rater composites of internalizing and externalizing symptom scores. These multi-rater composites for internalizing and externalizing symptom scores were correlated, *r* = .66, *p* = <.001. These scores were also mildly skewed, so we winsorized outliers to + 3 standard deviations of the mean and then used a square root transformation to normalize the distributions for later correlation analyses. This strategy successfully normalized the distributions (internalizing raw score: *M* = 6.57, *SD* = 5.88, skew = 1.53, kurtosis = 2.90, transformed score: *M* = 2.56, *SD* = 0.98, skew = 0.47 kurtosis = −0.41; externalizing raw score: *M* = 7.49, SD = 6.72, skew = 1.39, kurtosis = 1.86, transformed score: *M* = 2.69, *SD* = 1.08, skew = 0.46 kurtosis = −0.36).

**Figure 2.**
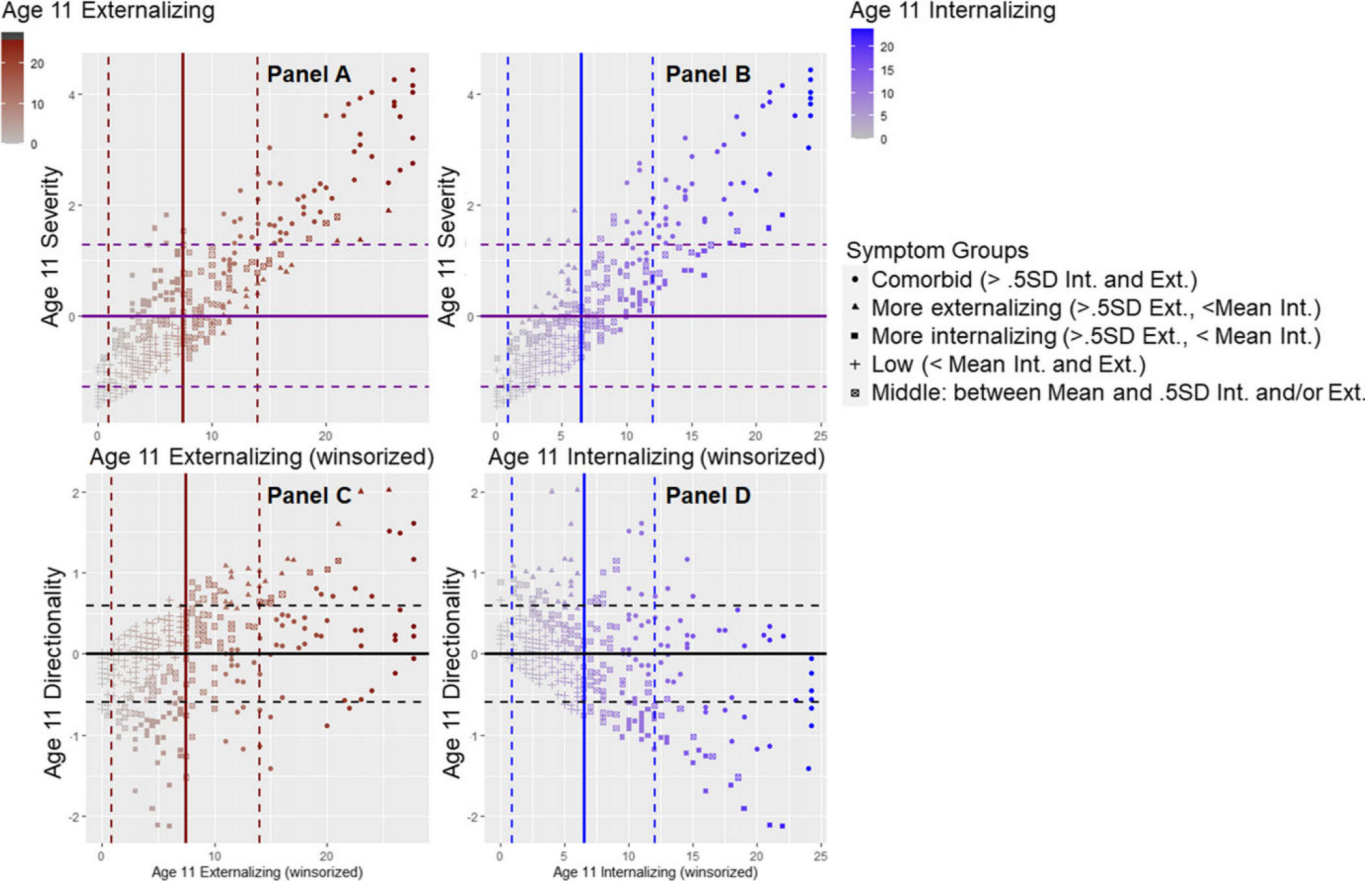
Psychopathology symptom severity and directionality. Heuristic showing relation between internalizing and externalizing symptom scores with severity and directionality scores. Thick lines indicate the sample mean for externalizing symptoms (red), internalizing symptoms (blue), symptom severity (purple) or symptom directionality (black). Using the same color scheme, hashed lines depict ± 1SD for the sample mean. The **top left panel (A)** shows that the majority of youth high in severity (e.g., above the 1SD line top purple hashed line) have comorbid problems (depicted by circles) and score above 1SD of externalizing symptoms (to the right of the right-most red hashed lines). Similarly, in the **top right panel (B),** youth high in severity have scores that are generally at the sample mean for internalizing symptoms, with very high severity also right of the ± 1SD line for internalizing symptoms. The **bottom left panel (C)** shows that youth scoring above 1SD in directionality tend to be youth with more purely externalizing symptoms who score>1SD on externalizing symptoms, but below the mean in internalizing symptoms. Circle and crossed-square points in this plot indicate youth who have comorbid symptoms, but show relatively more externalizing than internalizing symptom scores, and youth who are in the middle range of symptoms but have relatively more externalizing than internalizing symptoms scores. Likewise, the **bottom right panel (D)** shows that youth scoring below 1SD in directionality (more negative scores) tend to be youth with more purely internalizing symptoms who score>1SD on internalizing symptoms but below the mean in externalizing symptoms. Circle and crossed-square points in this plot indicate youth who have comorbid symptoms, but show relatively more internalizing than externalizing symptom scores, and youth who are in the middle range of symptoms but show relatively more internalizing than externalizing symptom scores. Individuals represented by + have below-average levels of both internalizing and externalizing symptoms, and indeed have scores of 0 or lower for symptom severity. These individuals also score near 0 for directionality. Notably, in the bottom plots, youth with comorbid problems also fall near the zero line for directionality, indicating youth with comorbid problems that are relatively balanced with regard to internalizing and externalizing symptoms.

Next, we subjected the internalizing and externalizing multi-rater composites to a Principal Components Analysis (PCA) extracting exactly two scores. We used the winsorized (but not square root transformed) multi-rater internalizing and externalizing symptom scores to maximize variability since the PCA is robust to skew that is in the same direction and magnitude for both variables. The first factor we extracted is symptom severity (eigenvalue = 1.65, explaining 83% of the variance, factor loadings = .91). Higher scores indicate more symptom severity regardless of type, with the highest levels indicating comorbid problems. The second factor is symptom directionality (explaining the remaining 17% of the variance, with externalizing symptoms loading .42 and internalizing symptoms loading −.42). Higher positive scores on directionality indicate more pure externalizing symptoms whereas more negative scores indicate more pure internalizing symptoms. Figure 2 depicts the relation between internalizing and externalizing symptom scores with symptom severity and directionality to aid in understanding and interpretation of these scores.

##### Genetic risk for psychopathology

The genetic risk scores were generated from data provided by birth mothers and birth fathers when available and have been previously described (Marceau et al., 2019). Within externalizing problems, internalizing problems, and substance use domains, scores were created from four types of indicators: (a) number of disorders on a diagnostic interview (listed below) (b) symptom counts (c) disorder onset age, and (d) proportion of first-degree relatives with that type of problem, as each of these phenotypes are expected to reflect greater genetic risk (see Marceau et al., 2019 for more details and specific items). For internalizing problems, indicators a-c came from the major depression, brief recurrent depression, dysthymia, separation anxiety, adult separation anxiety, social phobia, agoraphobia (with/ without panic), panic disorder, specific phobia, and generalized anxiety modules from the Composite International Diagnostic Interview (CIDI; Kessler & Üstün, 2004). For substance use, indicators a-c came from the alcohol abuse and dependence, drug abuse and dependence, and tobacco dependence modules from the CIDI. For externalizing problems, indicators a-c were based on the conduct disorder and antisocial personality modules from the Diagnostic Interview Schedule (DIS; Robins et al., 1981). Proportion of first-degree relative scores was asked via separate items for each domain.

As described by Marceau et al., 2019, there were several unique considerations for the BP data. First, birth parents were assessed on the diagnostic interview twice for the first cohort (*n* = 361) and only once for the second cohort (*n* = 200). We incorporated data from both assessments to utilize all available data and capture the best genetic risk for cohort 1 (see technical manual on osf.io for details). Second, if the onset of a disorder occurred at the age of conception or birth and not before or after, then it was not considered an indication of genetic risk, but rather a prenatal risk factor, and thus we set this data to 0 for the purposes of constructing this genetic risk score (this is important for applications wherein genetic and prenatal risk are disentangled, but less important for the current manuscript). Third, we made one modification to prior iterations of these scores: in the prior versions of the scores, we first imputed age of onset data such that if there was no age of onset for a symptom or disorder for all individuals they were given an age of onset of one year later than the oldest age of onset in the sample. However, in this iteration, we kept age of onset missing for birth fathers who provided no other data after we realized that we were unintentionally providing a relatively “less risky” value for age of onset for birth fathers with no other data, and therefore instead of imputing a more-or-less average score (in the absence of any information to improve prediction) we were assigning a below-average risk score for nonparticipating birth fathers. This limitation is corrected in the current version of the scores.^1^

##### Environmental risk for psychopathology

Adoptive parent (AP) psychopathology scores were created to index potential environmental risk for psychopathology. They were explicitly created to match BP (genetic risk) psychopathology scores as closely as possible, although there were some measurement differences. For internalizing problems, APs were not administered the agoraphobia (with and without panic) or separation anxiety (child or adult) modules, leading to a reduced item set used to construct indicators a-c compared to the genetic risk scores. In addition, DIS data were not collected for APs, so indicators a-c were unavailable for the externalizing domain. Instead, at child age 9 and 18 months, APs reported on their own antisocial behavior on the Antisocial Action Questionnaire (Levenson et al., 1995), which includes items such as telling lies, cheating at work or other places, stealing, and driving recklessly (conceptually similar to antisocial personality problems). The proportion of first-degree relatives and substance use modules were identical across BPs and APs. However, it is important to note that the meaning for interpretation of intergenerational effects of first-degree relatives of APs relative to BPs differs. For BPs, where mechanisms are genetic, more first-degree relatives indicate a likely higher genetic loading within the family as it pertains to child psychopathology. However, for APs, where transmission mechanisms are environmentally mediated, more first-degree relatives indicate a likely higher extended family shared environmental effect, as the adoptive parent and child would potentially have more exposure to family members with psychopathology.

##### Analytic strategy

All data were analyzed using R, version 4.3.0 (R Core Team, 2023). All code and results are available on https://osf.io/zx47e/?view_only=7729aef0ffd24322bc7132621d020a89.

##### Missing data

Missing data in the current study come from two sources: missing birth father data, and attrition. Although EGDS has possibly the largest sample of birth fathers in a developmental study, full birth father data are available for only for about 40% of the families. We examined differences in study variables and sample demographics across groups where (a) birth father data was completely missing (63%) vs. any data were provided, and (b) age 11 child psychopathology data was missing (26%) vs. was not missing. Variables included covariates and earlier predictors (cohort, sex, adoption openness, BP and AP psychopathology [for child psychopathology data only]), as well as demographic indicators (placement age, child race/ethnicity, AP1 and AP2 education, AP household income, birth mother, AP1 and AP2 ages at the birth of the adopted child). This yielded a total of 34 t-tests (Bonferroni-corrected α: 0.00147). Cohort 2 was more likely to be missing age 11 data than cohort 1, *t*(254.66) = −4.85, *p* < .0001. Adoptions that were more open were more likely to have birth father data than more closed adoptions, *t*(453.9.40) = 3.48, *p* < .001. See below for treatment of missing data.

##### Parent psychopathology scores

In brief, we used PCA, a data reduction technique well-suited to explain the maximal variance across multiple indicators, to create measures of externalizing problems, internalizing problems, and substance use based on four indicators of psychopathology (see below). PCA’s were computed in r using the FactoMineR (Lê et al., 2008) and missMDA (Josse & Husson, 2016) packages in order to accommodate missing data on the indicators with single imputation (see Marceau et al., 2019 for more detail). As a sensitivity analysis, we re-constructed scores that included data from birth fathers excluding missing data (listwise deletion) rather than using single imputation (see results section).

Supplemental Figure “Structure of Psychopathology Score” on the osf.io site depicts the various scores created: BM, BP, AP1, and AP2 scores for each domain were conditioned on the four indicators described in the measures section. Additionally, for more comprehensive measures, we also created BP and AP scores using the 8 indicators across both BPs and both APs respectively. The first (principal) component was saved as the factor score indicator for each parents’ psychopathology in each domain. Next, we ran a second series of PCAs where the indicators were the domain scores (e.g., BM internalizing problems factor score, BM externalizing problems factor score, and BM substance use factor score) which is akin to a p-factor. Once again, the first (principal) component was saved as the “p-factor” score, separately for BM, BF, AP1, AP2, and the BP and AP scores as well. Together, this yielded 12-factor scores capturing different aspects of potential environmental influences for psychopathology (AP1, AP2, AP * 3 domains + AP1, AP2, and AP *p*-factors) and 12-factor scores capturing different aspects of genetic risk for psychopathology (BM, BF, BP * 3 domains + BM, BF, and BP p-factors).

**Hypothesis testing** consisted of associations between the 24 factor scores and 4 outcome measures (child internalizing symptoms, externalizing symptoms, symptom severity, and symptom directionality). In these correlations, missing data were deleted listwise.

## Results

### Birth parent psychopathology scores

Eigenvalues, % variance explained, and factor loadings are presented in the top half of Table 1. Here we interpret factor loadings > .90 as very strong, .80–.89 as strong, .70–.79 as moderately strong, .40–.69 as moderate, and < .40 as weak, and < .20 as not loading. We expected disorder counts, symptom counts, and proportion of first-degree relatives with problems in the same domain to load positively on the factor scores, whereas age of onset to load negatively on the factor scores (since an older age of onset would indicate less genetic/overall psychopathology risk).

For internalizing problems, the BP score explained 36% of the variance common among all 8 indicators. BM and BF disorder counts loaded moderately strongly. BM and BF symptom counts, age of onset, and BM proportion of first-degree relatives with internalizing problems loaded moderately in the expected directions. However, BF proportion of first-degree relatives did not load on the overall internalizing problems factor score. BM and BF-specific scores explained about half of the variance among the 4 indicators for each individual, and factor loadings were strong for disorder and symptom counts, moderately strong for age of onset, and moderate (BM) or weak (BF) for proportion of first-degree relatives with internalizing problems.

For substance use, the BP score explained 53% of the variance common among all 8 indicators. BM and BF disorder and symptom counts loaded strongly in the expected direction. Age of onset and proportion of first-degree relatives with substance use problems loaded moderately in the expected directions for both BM and BF. BM and BF-specific scores explained nearly two thirds of the variance among the 4 indicators for each individual, and factor loadings were very strong for disorder and symptom counts, moderately strong for age of onset, and moderate for proportion of first-degree relatives with substance use problems.

For externalizing problems, the BP score explained 48% of the variance common among all 8 indicators. BF symptom counts loaded strongly, and BM and BF disorder counts and BM symptom counts loaded moderately strongly in the expected direction. BM and BF age of onset and BF proportion of first-degree relatives with externalizing problems loaded moderately in the expected directions, whereas BM proportion of first-degree relatives with externalizing problems weakly loaded on the externalizing factor. BM and BF-specific scores explained about half of the variance among the 4 indicators for each individual, and factor loadings were very strong for symptom counts, strong for disorder counts, and moderate for age of onset and proportion of first-degree relatives, all in the expected directions.

Eigenvalues, % variance explained, and factor loadings for the second-order “p-factors” are presented in the top half of Table 2. For BP, BM, and BF versions, the p-scores explained about 2/3 of the variance across domains. Substance use scores loaded strongly on the p-factor, as did externalizing problems for BM and BF scores (and nearly for BP), whereas internalizing problems scores loaded moderately strongly.

### AP psychopathology scores

Eigenvalues, % variance explained, and factor loadings are presented in the bottom half of Table 1. As above, in models for BP psychopathology scores, all factor loadings were in the expected directions.

For internalizing problems, the AP score explained 30% of the variance common among all 8 indicators. The strongest loadings were for AP2 disorder counts (strong), age of onset (moderately strong), and symptom counts (moderate). AP1 disorder and symptom counts and age of onset loaded moderately. AP1 and AP2 proportion of first-degree relatives with internalizing problems had weak loadings. AP1 and AP2 specific models were similar to each other, with strong (AP2) or very strong (AP1) loadings for disorder counts, moderately strong (AP2) or strong (AP1) loadings for symptom counts, and strong loadings for age of onset, although loadings for proportion of first-degree relatives with internalizing problems remained weak.

For substance use, the AP score explained 37% of the variance common among all 8 indicators. AP1 and AP2 disorder and symptom counts loaded moderately strongly, with moderate loadings for age of onset, and weak loadings for proportion of first-degree relatives with substance use problems. In AP1 and AP2 specific models, the disorder and symptom counts loaded very strongly, with strong loadings for age of onset, and still weak loadings for proportion of first-degree relatives with substance use problems.

For externalizing problems, AP scores were built on different types and numbers of (6 instead of 8) indicators yet explained a similar 30% of the variance. The two assessments of antisocial personality loaded moderately for AP1 and AP2, and the proportion of first-degree relatives with externalizing problems had similarl (to internalizing problems and substance use scores) weak loadings. In AP1 and AP2 specific models, the antisocial personality loadings were moderately strong, and somewhat stronger for 9 m relative to 18 m scores for both AP’s, with moderate loadings for proportion of first-degree relatives with externalizing problems.

Eigenvalues, % variance explained, and factor loadings for the second-order “p-factors” are presented in the bottom half of Table 2. For AP, AP1, and AP2 versions, the p-scores explained just under half of the variance across domains. For the AP combined and AP1 models, substance use and externalizing problem scores generally loaded moderately strongly on the p-factor, whereas internalizing problems scores loaded moderately. For the AP2 model, substance use loaded moderately strongly on the p-factor, followed by internalizing problems and then externalizing problem scores (both moderate loadings).

### Correlations

Generally, all associations for which the associated *p*-value was < .05 were small in magnitude. Associations for which *p* > .05 had effect sizes under *r* = .10 and can thus be considered too small to be meaningful. All correlation coefficients are presented in Figures. All results, including p-values, can be found on osf.io. Here we report the general pattern of effects.

#### Age 11 internalizing symptoms (Figure 3)

AP and AP1 internalizing problems were positively associated with child internalizing symptoms, seemingly supporting some homotypic environmental transmission. AP and AP1 p-scores were also positively associated with child internalizing symptoms, supporting some general environmental transmission. AP, AP1, AP, and BP externalizing problems were all positively associated with more child internalizing symptoms, supporting both environmental and genetic heterotypic transmission. No measures of parent substance use were associated with age 11 internalizing symptoms.

#### Age 11 externalizing symptoms (Figure 4)

AP, AP1, and AP2 externalizing problems were associated with child externalizing symptoms, seemingly supporting homotypic environmental transmission. No BP measures or AP measures of parent p-factor, substance use, or internalizing problems were associated with age 11 externalizing symptoms.

#### Age 11 symptom severity (Figure 5)

AP and AP1 p-scores were associated with age 11 comorbid symptom severity, indicating potential evidence of homotypic environmental transmission of comorbidity. AP and AP1 internalizing problem scores, and AP, AP1, and AP2 externalizing problem scores were also associated with age 11 comorbid symptom severity, indicating evidence of general environmental transmission for symptom severity. There was no evidence of genetic transmission. No measures of parent substance use were associated with age 11 symptom severity.

#### Age 11 symptom directionality (Figure 6)

Only BF p-score, substance use, and internalizing problems were associated with children having more internalizing than externalizing symptoms at age 11, weakly supporting general, heterotypic, and homotypic genetic transmission. There was no other evidence of intergenerational transmission of psychopathology influencing the specificity of problem type experienced by 11-year-olds.

#### Post-hoc sensitivity test

Because of the surprising findings regarding birth father psychopathology and adolescent symptom directionality, we restricted the sample to only families with birth father data. The effect sizes for the three findings became larger, *r* between −.17 and −.27, although the *p*-value increased to between *p* = .05 and .10. This change in *p*-value is a function of the *n* decreasing and standard errors increasing. In all, the pattern of findings suggests that these associations may reflect real associations not induced by treatment of missing data.

## Discussion

The present study adds to the literature on intergenerational transmission of psychopathology during adolescence by separating genetic and potential environmental mechanisms of transmission. This was accomplished by leveraging an adopted-at-birth design and investigating homotypic, heterotypic, and general transmission through the generation of multiple psychopathology scores for parents and adolescents. As hypothesized, potential environmental intergenerational transmission was somewhat stronger than genetic intergenerational transmission (e.g., correlations between AP’s and youth were generally stronger than correlations between BP’s and youth). This could be due in part to our study design, which is more powered to detect potential environmental influences than genetic transmission (see limitations section), however, this main finding is consistent with previous literature using different study designs. For example, Marceau et al. (2022) found no evidence of genetic transmission in a children-of-twins study of symptom severity and directionality, instead finding evidence for “phenotypic” transmission that could indicate either direct environmental influences from parent-to-child or gene-environment correlation (including evocative child effects).

**Figure 3.**
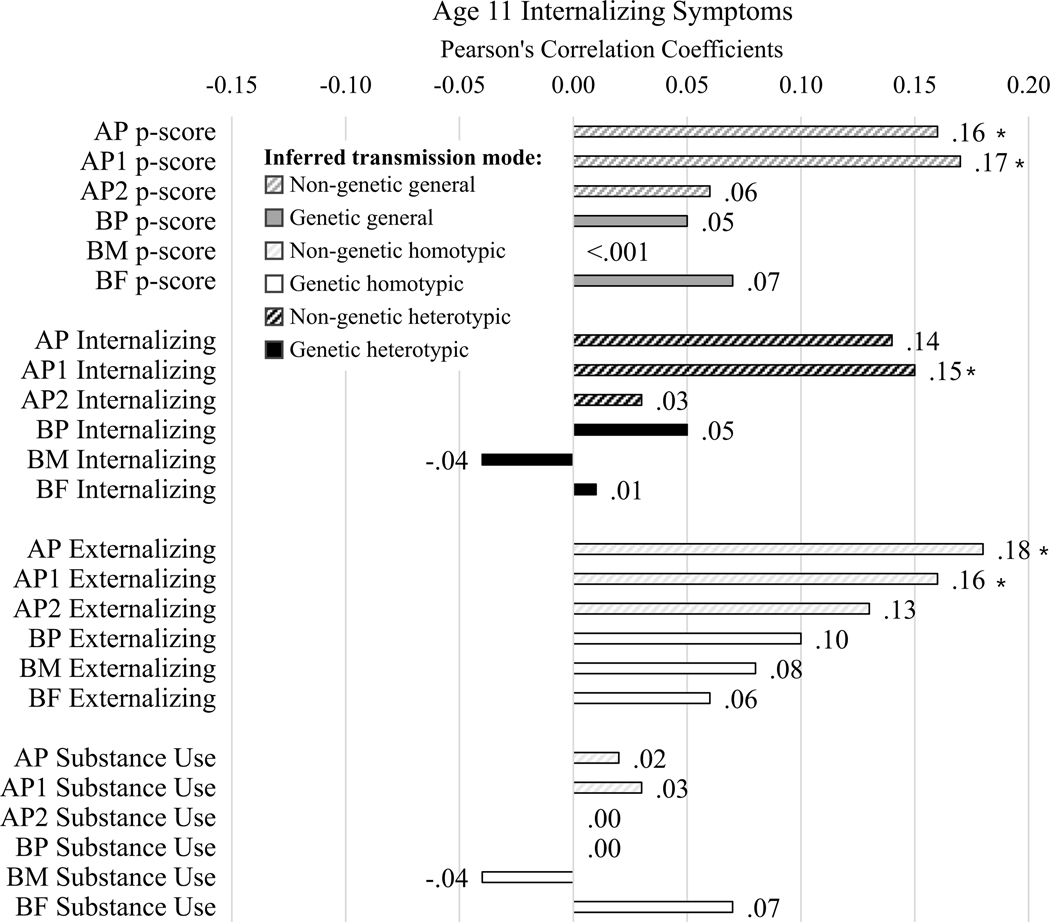
Correlation results for age 11 internalizing symptom scores. Diagonal line fill represents non-genetic transmission (correlations of AP and youth scores). Solid fill represents genetic transmission (correlations of BP and youth scores). Black fill color denotes evidence for homotypic transmission (e.g., correlations in the same domain, in this case parent internalizing because the child outcome is internalizing symptoms), white fill color denotes evidence for heterotypic transmission (e.g., correlations across domain, in this case parent externalizing and substance use because the child outcome is internalizing symptoms), dark gray fill color denotes evidence for general transmission (e.g., parent p-scores because the child outcome is internalizing symptoms). Bold Pearson’s coefficients highlight associations for which *p* < .05; asterisks note where associations would survive Benjamini-Hochberg correction for multiple testing.

**Figure 4.**
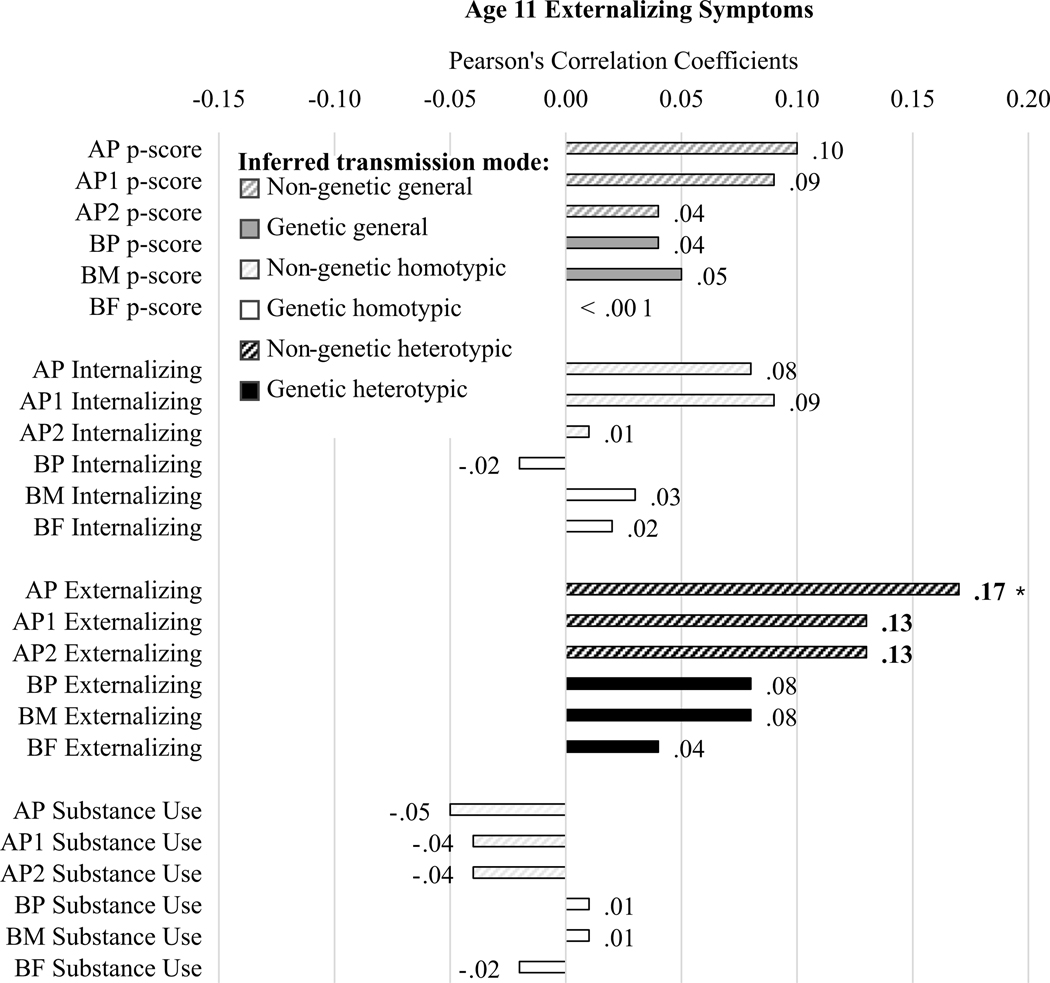
Correlation results for age 11 externalizing symptom scores. Diagonal line fill represents non-genetic transmission (correlations of AP and youth scores). Solid fill represents genetic transmission (correlations of BP and youth scores). Black fill color denotes evidence for homotypic transmission (e.g., correlations in the same domain, in this case parent externalizing because the child outcome is externalizing symptoms), white fill color denotes evidence for heterotypic transmission (e.g., correlations across domain, in this case parent internalizing and substance use because the child outcome is externalizing symptoms), dark gray fill color denotes evidence for general transmission (e.g., parent p-scores because the child outcome is externalizing symptoms). Bold Pearson’s coefficients highlight associations for which *p* < .05; asterisks note where associations would survive Benjamini-Hochberg correction for multiple testing.

**Figure 5.**
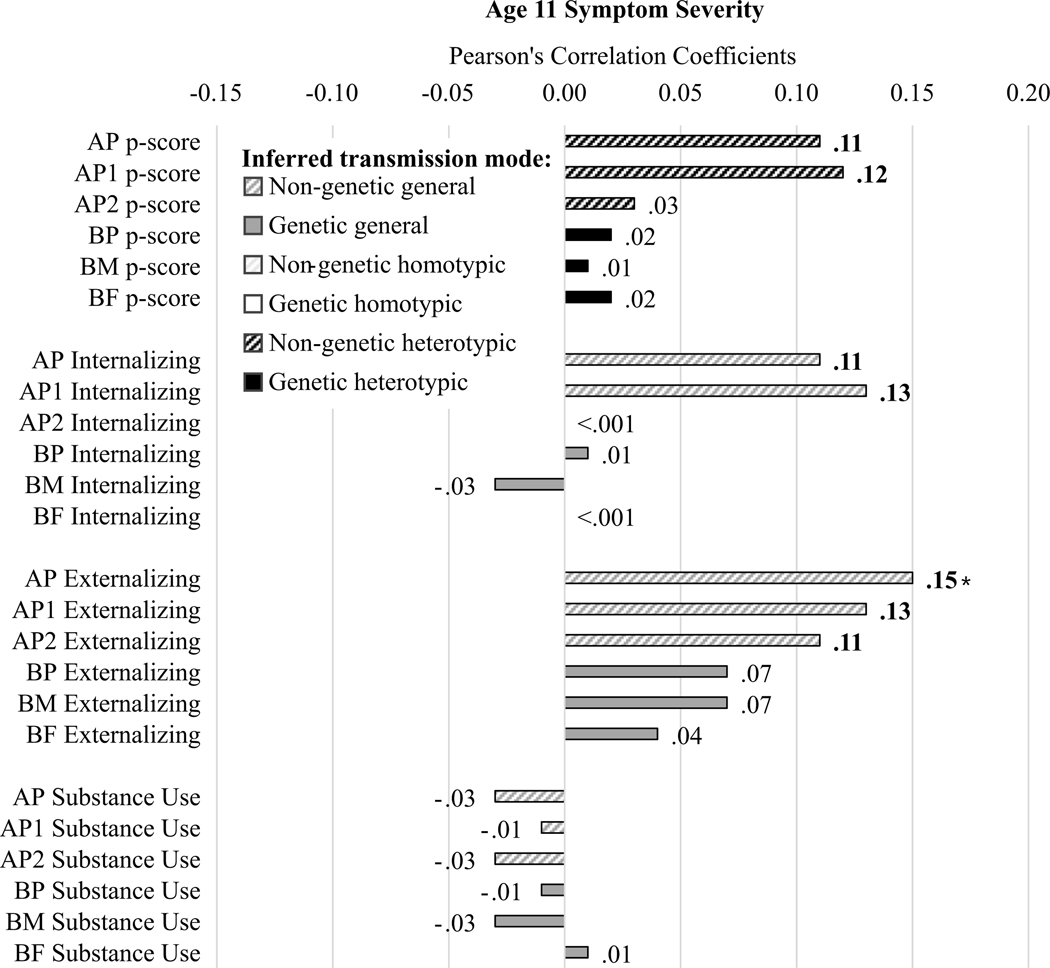
Correlation results for age 11 symptom severity scores. Diagonal line fill represents non-genetic transmission (correlations of AP and youth scores). Solid fill represents genetic transmission (correlations of BP and youth scores). Black fill color denotes evidence for homotypic transmission (e.g., correlations in the same domain, in this case parent p-scores because the child outcome is comorbid symptom severity), white fill color denotes evidence for heterotypic transmission (e.g., correlations across domain, not present because the child outcome reflects general psychopathology rather than domain-specific scores), dark gray fill color denotes evidence for general transmission (e.g., parent internalizing, externalizing, or substance use because the child outcome reflects general psychopathology). Bold Pearson’s coefficients highlight associations for which *p* < .05; asterisks note where associations would survive Benjamini-Hochberg correction for multiple testing.

**Figure 6.**
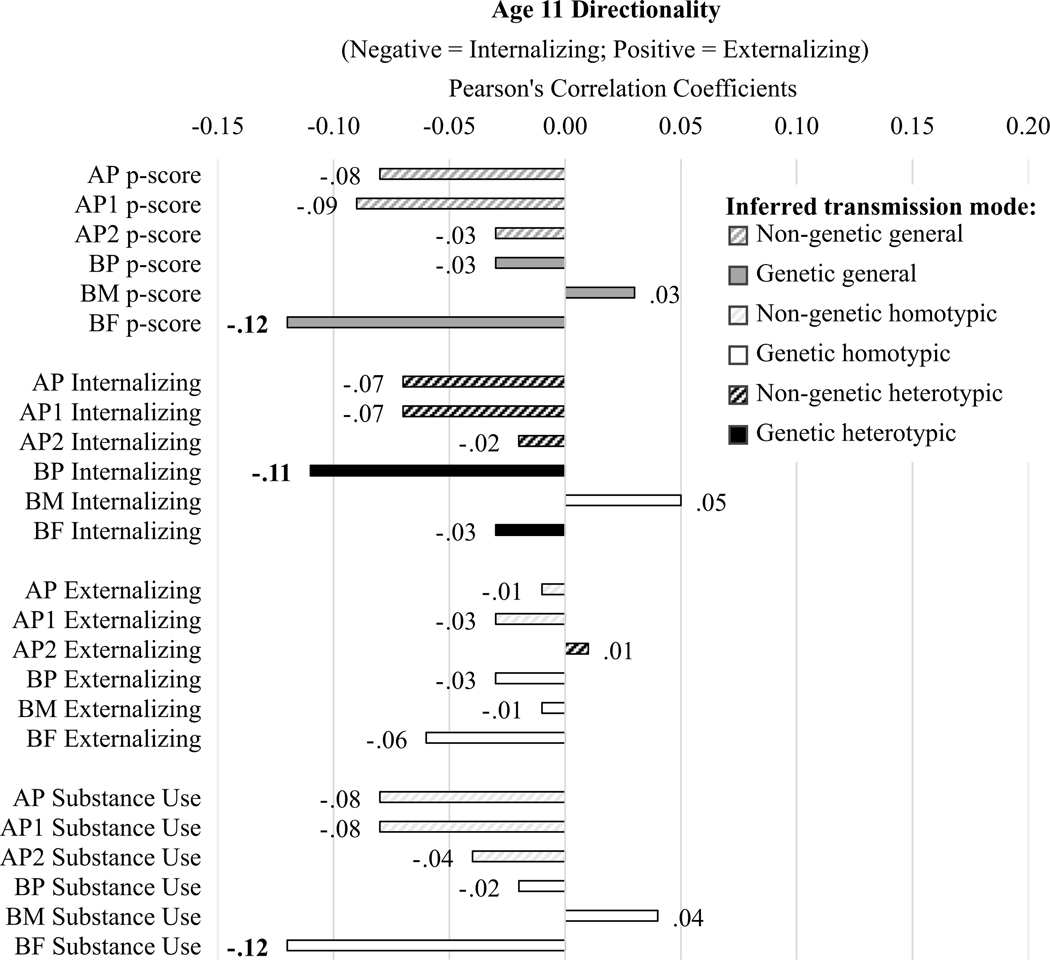
Correlation results for age 11 symptom directionality scores. Diagonal line fill represents non-genetic transmission (correlations of AP and youth scores). Solid fill represents genetic transmission (correlations of BP and youth scores). Black fill color denotes evidence for homotypic transmission (e.g., correlations in the same domain, in this case parent internalizing for negative associations but parent externalizing for positive associations since larger negative values indicate more purely internalizing symptoms but larger positive values indicate more purely externalizing problems in children), white fill color denotes evidence for heterotypic transmission (e.g., correlations across domain, in this case parent externalizing for negative associations but parent internalizing for positive associations since larger negative values indicate more purely internalizing symptoms but larger positive values indicate more purely externalizing problems in children), dark gray fill color denotes evidence for general transmission (e.g., parent p-scores because the child outcome is directionality, or domain-specificity of symptoms). Bold Pearson’s coefficients highlight associations for which *p* < .05; no associations survived Benjamini-Hochberg correction for multiple testing.

We expected to see some general and some homotypic transmission, with less evidence of heterotypic transmission. Our expectations were supported for general transmission and heterotypic transmission (e.g., across Figures 3−6 dark gray bars were generally somewhat larger, and white bars were somewhat smaller, and there were generally more and larger associations with symptom severity in Figure 5 than with symptom directionality – particularly white bars indicating heterotypic transmission – in Figure 6). However, the lack of support for homotypic transmission of internalizing and externalizing symptoms (e.g., few instances of large black bars in Figure 6, which would most strongly indicate homotypic intergenerational transmission) was surprising. This suggests that instances of homotypic transmission in the literature – particularly when studies do not account for comorbid problems across domains – may misinterpret the strength of homotypic transmission. Last, analyses yielded evidence of homotypic transmission of comorbidity assessed via associations of general psychopathology scores across generations (e.g., black bars in Figure 5), supporting our final hypothesis, albeit specifically for adoptive parents, especially AP1.

### Internalizing symptoms, externalizing symptoms, severity, and directionality

Our investigation of adolescent internalizing and externalizing symptoms, also in tandem with symptom severity and directionality revealed that intergenerational transmission does seem to operate via comorbid symptom severity, rather than by domain-specific symptoms. This evidence is consistent with prior investigations showing associations of symptom severity across generations (*r* = .29; Marceau et al., 2022). Although there was evidence of AP p-score and internalizing problem associations only with adolescent internalizing symptoms and not with externalizing symptoms, several sources of evidence suggest that this is not an interpretable difference. First, the effect sizes for associations between AP and AP1 p-scores and internalizing problems with adolescent internalizing symptoms (.14 to .17) were only slightly higher than for externalizing symptoms (.08 and .10) – this is unlikely to be a meaningful difference given that all effect sizes were already small. Second, AP, AP1, and AP2 externalizing problems associations with adolescent internalizing and externalizing symptoms were very comparable (.13 to .18 for child internalizing symptoms, .13 to .17 for child externalizing symptoms). Third, internalizing and externalizing symptoms scores were correlated *r* = .66 in this sample, indicating a high degree of symptom overlap or comorbidity. Fourth, findings from associations with symptom severity, which indexes the total level of problem severity regardless of type, and therefore also indexes comorbidity (because the highest levels of severity can only be reached by having both types of symptoms), entirely overlap with findings for both internalizing and externalizing symptoms.

Finally, none of the findings specific to adolescent internalizing or externalizing symptoms were replicated in the analysis with directionality – if they had been, this would indicate a specificity of influence. This was somewhat surprising, given evidence of some weak (*r* = .17) intergenerational transmission of symptom directionality in a children-of-twins study (Marceau et al., 2022). We did not measure symptom directionality in the parent generation here, as done in that study. There was some such evidence for homotypic transmission specifically for BF internalizing problems; however, we do not interpret these findings and urge extreme caution for readers thinking about this finding, as BF scores contained a lot of missing data, these are small effect sizes, and BF p-score and substance use domain scores also associated with a preponderance of adolescent internalizing symptoms. The idea that genetic influences from the paternal line may have a specific influence on the intergenerational transmission of internalizing symptoms is intriguing but the data presented here are not strong enough to do more than potential hypothesis generation for other studies with fewer data limitations with respect to the paternal genetic line to test. In all, our evidence most strongly supports the general transmission, particularly potentially environmental transmission of psychopathology that is likely to put youth at risk of developing comorbid internalizing and externalizing symptoms.

### Genetic, environmental, and evocative transmission

Our results suggest that the caregiving (adoptive) parents’ psychopathology scores were more correlated with adolescent psychopathology than birth parents’ psychopathology scores. This could indicate genetic transmission is less potent than environmental transmission. This could also indicate reverse transmission, such that parents’ symptoms are evoked responses to children’s internalizing and externalizing symptoms. A recent study found that in Dutch families, there were between-family associations such that mothers’ but not fathers’ stable internalizing and externalizing symptoms were associated with adolescents’ internalizing and externalizing symptoms (correlations of random intercepts of the scores repeatedly measured across adolescence). Within families, the only lagged effects were from increases in adolescent internalizing symptoms to increases in maternal internalizing symptoms a year later across adolescence, suggesting that homotypic transmission of internalizing symptoms may be more potent for mothers than fathers, and actually reflect evocative processes (Schulz et al., 2021). Although our study assessed parent psychopathology a few years preceding the assessment of child internalizing and externalizing symptoms, given stability in psychopathology over time, it is possible that earlier evocative effects unmeasured in this analysis may contribute to the current findings.

### Implications for prevention and intervention

As the current findings were consistent with general rather than specific transmission and suggested environmental rather than genetic influence (albeit the potential for evocative genetic influence is still possible) in the development of comorbid internalizing and externalizing symptoms during adolescence, they offer a couple of implications for the development and application of preventions and interventions. First, the results replicate the importance of assessing parenting and parent-youth interactions in assessing risk for adolescent co-occurring problem behavior. Furthermore, because of the use of a genetically sensitive research design, the study’s findings offer unique validation of the salience of incorporating parental influences in the assessment of youth comorbid problem behavior, as most prior research in this area has not been able to partition environmental from passive genetic influences (Gilliom & Shaw, 2004; Hentges et al., 2020). Second, consistent with our findings about general versus specific transmission, recent work on understanding the mechanisms underlying improvements in adolescent mental health and substance use from family-based interventions like the Family Check-Up suggest that the vast majority of variance in such changes is accounted for by a shared general “p” factor (Tein et al., 2023). These findings are consistent with the current study’s in suggesting the importance of focusing on the severity of youth symptoms across externalizing and internalizing domains, rather than continuity within specific problem behavior types.

### Limitations and special considerations

A primary limitation of inference in the current study is that genetic risk in the adopted-at-birth design is inferred based on associations between birth parent characteristics and adopted child characteristics. Although we strove for as similar measures for adoptive parents and birth parents as possible, there were some differences in measurement across birth and adoptive parents. Thus, in this study, null effects for genetic transmission, unfortunately, cannot imply that genetic influences are less important, but instead may indicate a combination of measurement error or a mismatch in birth parent and adopted child phenotypes – not only in terms of measurement strategy but also potentially due to age differences between the adoptee and the birth parent and the development of the phenotype. Further, psychopathology symptoms and diagnoses, while heritable, are not explained completely by genetic influences. This means that much of the psychopathology endorsed by birth parents is likely to be contextual or driven by environmental influences that the birth parents do not pass on to the adopted child. The strength of the genetic intergenerational transmission is bounded by the extent to which the birth parents’ psychopathology scores are driven by genetic influences and more specifically genetic influences they also passed onto the child. Thus, this design is far more powerful for understanding postnatal environmental and evocative processes in intergenerational transmission of psychopathology than it is for revealing genetic transmission. Other genetically informed designs are needed to better examine genetic transmission.

Another consideration in the measurement of parent psychopathology is that we used “lifetime” measures occurring prior to the assessment of adolescent psychopathology. This strategy loses some important developmental information as symptoms in parents will have fluctuated over time, and the timing of periods of parents’ symptoms may have influenced the developmental trajectory of youths’ psychopathology symptoms idiosyncratically across the sample. This loss of developmental information is not likely to systematically bias the current findings but may have reduced the general effect sizes with respect to intergenerational transmission, as we are relying only on the stable effects observable between lifetime parent psychopathology and age 11 youth psychopathology. That is, we cannot speak to developmental timing or course in this study.

The second major limitation of the study is the potential of shared method variance inflating associations between caregiver and child psychopathology. Adoptive parents reported on their own psychopathology symptoms and adolescent psychopathology symptoms. This could result in shared method variance for multiple reasons: First, individuals may gravitate towards certain parts of a scale and similarly answer all question types in a way that would contribute to similarities between scores. This is unlikely given the very different measurement strategies between adoptive parent phenotypes and the Child Behavior Checklist. Further, we attempted to mitigate this shared method variance limitation by examining AP1 and AP2 separately as well as combining both parents to form psychopathology scores via principal components analysis but using combined rater composites (averages) for adolescent externalizing symptoms. Further, there was quite a bit of time between the assessments of AP psychopathology (drawing from data collected when the child was 9 and 18 months and 6 or 8 years old) and the assessment of adolescent psychopathology (at age 11 years). In addition, parents’ own symptoms may color the way they view their children’s psychopathology. A recent study examined whether concordance between parent- and youth-reported psychopathology differs for parents with current (vs. remitted) depressive symptoms – all parents had a history of depressive symptoms which controls to some extent for genetic intergenerational transmission in that sample (Löchner et al., 2024). They found differences such that depressed parents were more likely to report more elevated psychopathology in youth compared to youth reports, although this effect was specific to diagnostic instruments rather than the Child Behavior Checklist used here, mitigating this concern for the current study. Shared method variance would inflate associations in the current study. The use of different strategies per measure should attenuate shared method variance, and because correlations were quite small, we believe this bias to be appropriately mitigated. In the future, it will also be important to incorporate youth self-report into the study of young adolescent problem behaviors. There are well established informant discrepancies in youth psychopathology assessment, each contributing valuable information (e.g., De Los Reyes et al., 2023). Multi-informant ratings across youth and parent reports were not included here based on measurement differences and challenges to harmonize such measures. Thus, future work carefully considers how best to harmonize across measures and form multi-rater composites to address commonalities and advance this line of research (De Los Reyes et al., 2023).

The third major limitation was missing data, particularly on the birth fathers. Birth fathers are an incredibly under-represented population in developmental science and a very hard-to-reach (or know) population. Thus, we believe that it is imperative to include any data we have, regardless of how much missing data there may be. We have chosen to use imputation procedures to handle the missing data in psychopathology score construction, although still not ideal, based on evidence that this remains a better choice than listwise deletion (Blozis et al., 2013). This choice, we believe, led to some findings we purposefully did not discuss that may be tempting to interpret. Namely, the BP scores, across domains, explained about 10–15% more of the common variance across indicators than the corresponding AP scores. One may be tempted to interpret that the birth parent population may have higher rates of assortative mating on psychopathology than the adoptive parent population; however, we believe that since BM scores on the indicators were to some extent used to impute BF scores in the combined BP model, this imputation procedure likely inflated the commonalities. Indeed, when assessing the commonality across psychopathology indicators in the scores drawing from single individuals, the BM/BF-specific scores match much more closely to the AP1/AP2 specific scores. The 10–15% increase in variance explained in the BM/BF compared to BP models, and ∼ 20–30% increase in variance explained in the AP1/AP2 compared to AP models is because the psychopathology indicators of one individual have more in common than the psychopathology indicators of two individuals. The only interpretation we can make about assortative mating is that since the BP and AP scores did have a substantial amount of common variance and high factor loadings across both individuals, there is evidence across both populations of assortative mating, and/or that psychopathology converges over time (e.g., lateral transmission of psychopathology among mated couples).

## Conclusions

Reducing adolescent psychopathology symptoms is a critical developmental goal given the high burden for adolescents experiencing these symptoms and the risk even sub-clinical symptoms convey for life-long problems with psychopathology and substance use. Understanding how psychopathology is transmitted across generations may help to clarify the types of prevention and intervention efforts leveraged to break the cycle. Our results most strongly support the general transmission, particularly via environmental and/or evocative rather than genetic mechanisms, and especially for AP1 (who predominantly identify as mothers). Future studies leveraging EGDS data may yet reveal whether in this sample these associations reflect a parent-to-child process that is likely to put youth at risk of developing comorbid internalizing and externalizing symptoms, or whether youth with comorbid internalizing and externalizing symptoms strain the mental health of caregivers, and potentially especially mothers.

## Supplementary Material

1

## Figures and Tables

**Table 1.** First order, domain-specific PCA results

			Factor Loadings							
	Eigenvalue	% Variance Explained	BM Dx count	BM Sx count	BM age of onset	BM 1^st^ deg. Rel.	BF Dx count	BF Sx count	BF age of onset	BF 1^st^ deg. Rel.

BP Internalizing	2.87	36%	.71	.67	−.67	.19	.74	.53	−.53	.51

BM Internalizing	2.12	53%	.80	.81	−.74	.52	–	–	–	–

BF Internalizing	2.02	50%	–	–	–	–	.83	.84	−.76	.23

BP Substance Use	4.21	53%	.80	.82	−.67	.57	.84	.86	−.67	.45

BM Substance Use	2.52	63%	.91	.91	−.77	.52	–	–	–	–

BF Substance Use	2.46	62%	–	–	–	–	.91	.90	−.77	.49

BP Externalizing	3.82	47%	.78	.82	−.53	.33	.82	.85	−.65	.56

BM Externalizing	2.22	55%	.87	.92	−.67	.41	–	–	–	–

BF Externalizing	2.25	55%	–	–	–	–	.89	.92	−.61	.48

	Eigenvalue	% Variance Explained	AP1 Dx count	AP1 Sx count	AP1 age of onset	AP1 1^st^ deg. rel.	AP2 Dx count	AP2 Sx count	AP2 age of onset	AP2 1st deg. rel.

AP Internalizing	2.42	30%	.42	.48	−.42	.21	.80	.67	−.77	.36

AP1 Internalizing	2.33	58%	.91	.81	−.87	.27	–	–	–	–

AP2 Internalizing	2.14	53%	–	–	–	–	.87	.77	−.83	.30

AP Substance Use	2.97	37%	.75	.73	−.63	.29	.71	.77	−.49	.25

AP1 Substance Use	2.52	63%	.96	.92	−.81	.26	–	–	–	–

AP2 Substance Use	2.58	65%	–	–	–	–	.95	.91	−.85	.36

			AP1 9m antisocial	AP1 18m antisocial	AP1 1^st^ deg. rel.	AP2 9m antisocial	AP2 18m antisocial	AP2 1^st^ deg. rel.

AP Externalizing	1.81	30%	.58	.57	.36	.66	.69	.34

AP1 Externalizing	1.38	46%	.79	.70	.51	–	–	–

AP2 Externalizing	1.49	50%	–	–	–	.83	.73	.53

*Note:* BP = birth parents; BM = birth mother; BF = birth father; AP = adoptive parents; AP1 = adoptive parent 1; AP2 = adoptive parent 2; Eigenvalue and % variance explained are presented for the first principal component (the saved factor score). Dx count = the number of disorders qualified for on a diagnostic interview across disorders in that domain; Sx count = the count of symptoms endorsed on a diagnostic interview across disorders in that domain; 1^st^ deg. rel. = proportion of first-degree relatives with problems in that domain. 9m antisocial = scores on the antisocial action questionnaire when the adopted child was 9 months old. 18m antisocial = scores on the antisocial action questionnaire when the adopted child was 18 months old. – indicates that the indicator was not used in the score creation for that row.

**Table 2.** Second order, p-factor PCA results

	First principal component statistics		Factor Loadings		
	Eigenvalue	% Variance Explained	Internalizing score	Substance Use score	Externalizing score

BP	1.89	63%	.74	.86	.78

BM	1.90	63%	.72	.85	.81

BF	2.03	68%	.79	.86	.82

AP	1.32	44%	.57	.70	.72

AP1	1.29	43%	.56	.69	.71

AP2	1.40	47%	.69	.76	.59

## Data Availability

Data supporting the findings of this study are available upon reasonable request to the study PIs (Ganiban, Leve, and/or Neiderhiser). All data prep and analytic code and results are available via the tech report available at https://osf.io/zx47e/?view_only=7729aef0ffd24322bc7132621d020a89.

## Footnotes

1 Analyses conducted before and after this correction, available upon author request, yielded no meaningful difference in patterns of findings.
