# Supplementary material for: Intergenerational transmission of comorbid internalizing and externalizing psychopathology at age 11: Evidence from an adoption design for general transmission of comorbidity rather than homotypic transmission
Source: Dev Psychopathol. Author manuscript; Available in PMC 2025 Jul 8. (PMC11968446; doi:10.1017/S0954579424000968)
Supplement: 1 [file NIHMS1987196-supplement-1.docx]

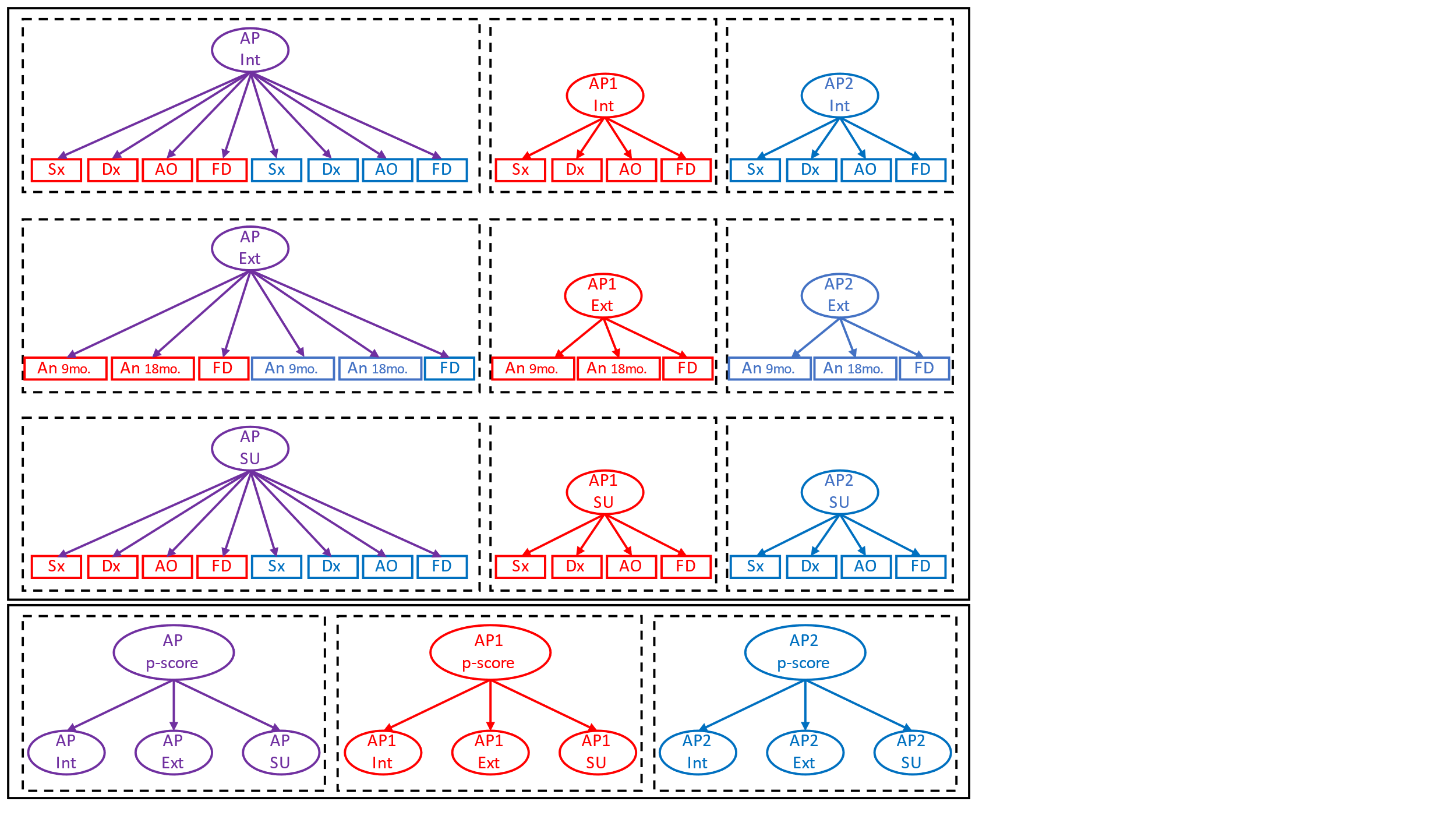

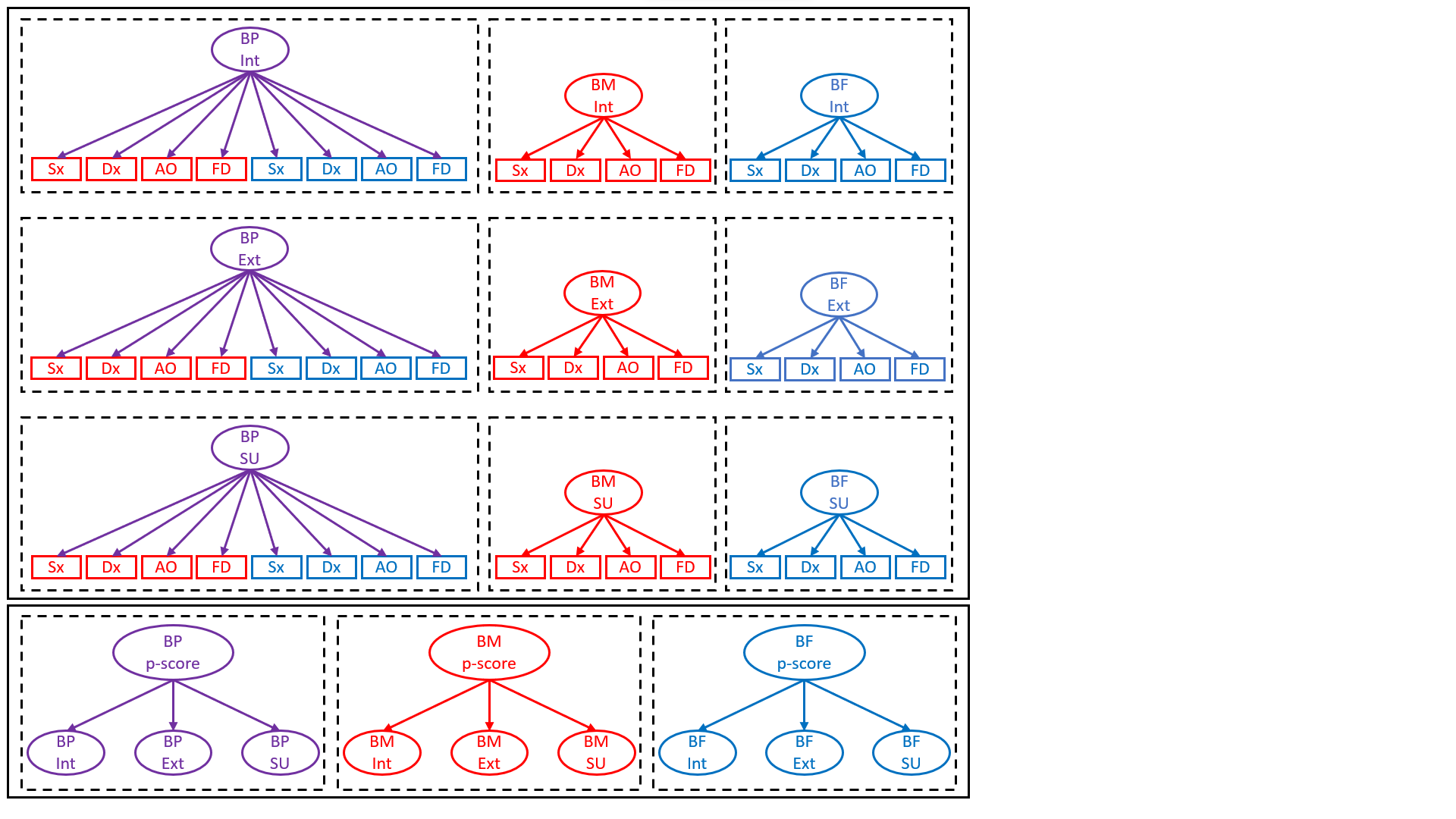


**This figure depicts the various parent psychopathology scores created for EGDS.**

*Note.* The black-outlined boxes on the left depict adoptive parent (AP) scores. There are two adoptive parents: AP1 (usually but not always mothers), and AP2 (usually but not always fathers). Birth parents include birth mothers (BM) and birth fathers (BF) when available.

The black outlined boxes on the right depict birth parent (BP) scores.

Additional Abbreviations: Symptom Count (Sx), Diagnoses Count (Dx), Age of Onset (AO), First Degree Relatives (FD), An (Antisocial Personality).

Color description: Purple indicates measures across two parents (AP1/AP2 or BM/BF); Red indicates AP1 or BM measures; Blue indicates AP2 or BF measures.

Each PCA analysis was done in 2 steps (Step 1: subscale, first three rows; Step 2: p-score, bottom row); Separate analyses was done for row 1 (internalizing scores), row 2 (externalizing scores), row 3 (substance use scores), and row 4 (p-scores).
